# Skin Cancer Awareness, Beliefs, Behaviors, and Access to Care Among the Homeless and Underinsured Patients Using a Free Clinic in Nebraska

**DOI:** 10.7759/cureus.74447

**Published:** 2024-11-25

**Authors:** Michelle S Swedek, Mariah Fleischman, Alexander Hall, Bruce Houghton

**Affiliations:** 1 Department of Medicine, Creighton University School of Medicine, Omaha, USA; 2 Department of Biostatistics, Creighton University School of Medicine, Omaha, USA

**Keywords:** dermatology, free clinic, health inequities, homeless, skin cancer awareness, skin neoplasms, student run clinic, sun protection factor, uninsured population

## Abstract

Introduction: To understand the attitudes, beliefs, knowledge, and access to care surrounding sun safety for a primarily homeless or underinsured patient population at a student-run health clinic.

Methods: All adult attendees at the health clinic were invited to complete an anonymous 16-item questionnaire that assessed their sun safety history, practices, knowledge, and beliefs.

Results: Fifty participants completed our questionnaire, with 35 individuals (70%) reporting that they were without permanent residence, and 21 individuals indicating that they were uninsured or using Medicaid (42%). The typical reported daily sun exposure was high (median=4 hours), while sunscreen use was uncommon (mean=1.83, falling between a “Never” or “Rarely” rating). Only 10 participants (20%) reported having ever seen a dermatologist though commonly assumed barriers such as transportation, cost, or wait time were not endorsed at high rates.

Conclusion: Our findings highlight areas for targeted education surrounding sun safety and identify sun protection methods accessible for this patient population.

## Introduction

Despite public health initiatives, the incidence of skin cancer continues to rise in the United States (US) [1]. Basal cell carcinoma (BCC), squamous cell carcinoma (SCC), and melanoma are the three most common types of skin cancer and collectively constitute 99% of the skin cancers in the US [1]. Between 1990 and 2019, the incidence of melanoma per 100,000 persons increased from 12.6 to 17.0, and its prevalence increased from 99.5 to 138 in the country [2]. In this same timeframe, the incidence of non-melanoma skin cancers (including both BCC and SCC) per 100,000 persons increased from 402 to 787, while their prevalence increased from 294 to 359 [2]. While genetic factors play a role in skin cancer susceptibility, ultraviolet (UV) exposure is a significant and modifiable risk factor for these three cancers [1]. It is well-known that exposure to UV wavelengths can induce direct cell damage and modulate immune function [3]. Most of these cases can be prevented with adequate sun protection and by minimizing UV exposure [1]. Beyond modifying dangerous exposure, skin cancer screening by primary care physicians and dermatologists can detect suspicious lesions earlier and prevent or mitigate skin cancer.

Unfortunately, individuals who are most likely to benefit from adequate sun protection and early dermatologic care (those who are less able to avoid extensive exposure) are often underserved - such as those with historically underserved backgrounds, the uninsured or underinsured, and those who are homeless. In Nebraska, it’s estimated that 8% of the population is uninsured, while 16% has Medicaid insurance [4,5]. Medicaid and uninsured patients are estimated to make up around 25% of the US population. They are underrepresented in dermatology practices and only comprise 5% of dermatology patients nationally, which reflects possible access barriers for these patients to receive dermatologic care [6]. Further, prior work has highlighted that patients without a permanent residence may have a higher incidence of skin cancer compared to those with a residence, most likely caused by increased sun exposure and decreased access to care [7]. In the Omaha and Council Bluffs Continuum of Care, an estimated 1,475 individuals experienced homelessness every night and 13.9% of them were completely unsheltered [8]. Importantly, as compared to White individuals, non-White individuals are more likely to present with later-stage skin cancer at diagnosis [9]. Individuals of historically underserved backgrounds are disproportionately overrepresented amongst Nebraska's homeless population. In Nebraska, 26.8% of homeless individuals are Black, despite making up 5.5% of the total state population [8,10]. Given these statistics, the homeless population of Nebraska is especially at risk for serious dermatologic conditions compared to the general population, while simultaneously being the least likely to receive care.

Inequity in dermatology care has been attributed to several causes, including insufficient number of dermatologists, long wait times, and practices limiting the number of Medicaid/uninsured patients that can be seen [6,11,12]. Free clinics are an important safety net in the US healthcare system that often provide healthcare to traditionally underserved populations [13]. These clinics may be the first place a patient presents for a skin cancer concern. Given the numerous barriers to dermatologic care and the heightened risk of skin cancer in this population, it is important to understand what obstacles and knowledge gaps may exist in the patients’ ability to obtain this care. By understanding these issues, free clinics may be better equipped to provide dermatologic screenings and education. To our knowledge, no study so far has described the state of skin cancer and sun safety knowledge amongst the homeless and underinsured populations in the Midwest US. To address this gap, the objectives of this study were to evaluate the patients' ability to access dermatologic care and skin cancer screenings, and to describe the knowledge, perspectives, behaviors, and reported barriers to care for skin cancer in a primarily homeless and underinsured patient population. In so doing, we aim to better understand our patient population’s knowledge about sun safety to identify areas for greater patient education.

## Materials and methods

Setting and participants

This cross-sectional survey was conducted at a student-run clinic during a health fair at an emergency homeless shelter in Omaha, Nebraska. This annual health fair provides screening and urgent care services free of charge to a diverse patient population experiencing homelessness or lacking insurance. The services provided include a consultation with a primary care physician, blood pressure screening, glaucoma screening, testing for sexually transmitted infections, and legal services to help participants sign up for insurance and social services. Approximately 200 individuals participated in the health fair. The inclusion criteria were English-speaking adults aged 19 years or older (the age of majority in Nebraska) who had reviewed the study information letter. Exclusion criteria included non-English-speaking individuals, as well as those who were working or volunteering at the health fair. This study was deemed exempt by the Creighton University Institutional Review Board (reference no. 2004203).

Questionnaire design

A 16-item questionnaire was developed to assess the patients’ perceptions of dermatologic risk, care, sun-safety behaviors, and barriers to visiting a dermatologist. These items were adapted from an open-access survey that had been previously validated. Notably, it included a survey piloted for readability and accessibility amongst individuals at a homeless shelter by Joseph et al. [14-17]. The questionnaire developed by the authors is available in Appendix 1 and includes the categories of questions, as well as correct responses to questions regarding knowledge. Question formats included binary response items such as “Do you feel that you sunburn easily?” and “Have you ever seen a dermatologist?”; multiple-choice questions like “When should sunscreen be applied for best protection?”; true/false knowledge questions such as “Having a tan protects my skin from the sun”; and Likert-type scale prompts, for example, “I am at risk for skin cancer” (rated on a five-point scale as 1=Strongly Disagree, 2=Somewhat Disagree, 3=Neutral, 4=Somewhat Agree, and 5=Strongly Agree). The responses regarding sun safety practices were reported in a Likert-type scale format with a similar scale [1=Never (0% of the time), 2=Rarely (25% of the time or less), 3=Sometimes (about 50% of the time), 4=Often (75% of the time or more), and 5=Always (100% of the time)]. Participants were also asked to identify their skin tone using a color bar scale based on an adapted version of the open-access six Fitzpatrick skin type categories [18]. In total, six prompts reflected sun safety knowledge, six reflected sun safety practices, and four reflected sun safety attitudes. These three categories were reported as mean responses with a 95% boot-strapped confidence interval (BSCI). Information regarding age, race, ethnicity, and insurance and housing status was also collected. 

Analysis

This was a descriptive study. Depending on data distribution, statistics were presented as mean and standard deviation (SD) or median and interquartile range (IQR) for continuous variables, and as frequency and percentage for categorical variables. Analyses were conducted using IBM SPSS Statistics for Windows, Version 28 (Released 2021; IBM Corp., Armonk, New York, United States).

## Results

A total of 50 participants agreed to complete the study questionnaire, with 35 (70%) indicating they were without permanent residence and 38 (76%) stating that they were uninsured or on Medicaid/Medicare. The “other” group was used for those of racial identities not otherwise mentioned or if patients were identifying with more than one racial identity. No participants identified themselves as of Asian racial origin (Table 1).

**Table 1:** Demographic characteristics of the participants (n=50) N: frequency; %: percentage of total cohort

Demographic characteristic	N (%)
Race	
White	25 (50%)
Black	13 (26%)
Other	4 (8%)
Not specified	8 (16%)
Ethnicity	
Hispanic/Latino	8 (16%)
Other than Hispanic/Latino	29 (58%)
Not specified	13 (26%)
Insurance	
Insured (Medicare/Medicaid/Private)	38 (76%)
Private	17 (34%)
Medicare alone	6 (12%)
Medicaid alone	8 (16%)
Medicare and Medicaid	7 (14%)
Uninsured	6 (12%)
Missing	6 (12%)
Residence	
Permanent residence (Own/rent home)	8 (16%)
Without permanent residence (live with friends/family, shelter, homeless/no fixed residence)	35 (70%)
Missing	7 (14%)
Fitzpatrick skin type	
1	18 (36%)
2	5 (10%)
3	3 (6%)
4	2 (4%)
5	2 (4%)
6	3 (6%)
Missing	17 (34%)

The median time spent outside in the summer months was four hours daily (IQR 2.00-5.25). Results concerning perceptions of risk and sun safety attitudes are available in Table 2. A minority of patients (n=21; 42%) indicated that they sunburn easily, and only 10 (20%) endorsed ever having seen a dermatologist. 

**Table 2:** Risk perception and sun safety A total of 50 individuals responded. N: frequency of "yes" response; %: percentage of total cohort

Question	N (%)
Do you feel that you sunburn easily?	21 (42%)
Have you ever seen a dermatologist for any reason?	10 (20%)
Have you ever had your skin checked head to toe for skin cancer by a dermatologist or other health professional?	7 (14%)
What is the most significant barrier you have encountered in obtaining dermatologic care? (select one)	
Transportation limitations	8 (16%)
Insurance/cost concerns	8 (16%)
Long wait times for appointments	2 (4%)
None of the above	32 (64%)

Regarding barriers to care, 18 (36%) patients selected transportation, cost, or appointment wait time as the primary barrier, with the remaining 32 (64%) patients indicating “none of the above.” The six-item general sun safety knowledge [mean=3.20, 95% BSCI (2.84-3.57)] and the six-item reported sun safety practice [mean=2.52, 95% BSCI (2.33-2.72)] scores were generally low. However, findings for the four-item sun safety attitudes measure were notably higher [mean=3.80, 95% BSCI (3.56 - 4.01)]. The most reported sun protection habit (five-point scale) was wearing a shirt with sleeves that cover the shoulders, (mean=3.5) and the least common was wearing clothing made of specific sun-protective material (mean=1.7) (Table 3). 

**Table 3:** Sun protection habits A total of 47 responses were collected for this portion. SD: standard deviation

Prompts	Mean (SD)
How often do you wear sunscreen?	1.83 (1.09)
How often do you wear a shirt with sleeves that cover your shoulders?	3.45 (1.27)
How often do you wear a hat?	2.60 (1.53)
How often do you stay in the shade or use an umbrella?	2.45 (1.33)
How often do you wear sunglasses?	2.94 (1.36)
How often do you wear clothing made with specific sun protective material?	1.70 (1.16)

## Discussion

This study assessed sun safety access, practices, competency, and attitudes at an emergency shelter for the homeless in Omaha, Nebraska. With an increased propensity for developing precancerous and cancerous lesions due to increased sun exposure, limited access to sun protective resources, and constrained healthcare accessibility, the homeless population requires considerable attention in the context of the national increase in the incidence of skin cancer [7]. Our initiative identified knowledge gaps surrounding tanning, sun protection, and skin cancer warning signs. We also saw high-risk skin cancer behaviors, including significant time spent outside, amongst the homeless and underinsured populations seen at our student-run clinic at the health fair.

A baseline history of severe sunburn has been strongly correlated with an increased risk of SCC, BCC, and melanoma [19]. Since 21 participants (42%) in the survey reported susceptibility to sunburn, sun avoidance should be considered essential in mitigating skin cancer risk. While the time to sunburn depends on the skin type, individuals with light-to-medium toned skin (Fitzpatrick type I and type II) can expect sunburn after 20 to 60 minutes of exposure to UV radiation, depending on its index [20]. In the summer months, Nebraska experiences an average UV index of 8.5, suggesting that individuals of type I and type II are likely to experience sunburn after just 20 minutes of sun exposure, while those classified as type III and type IV may experience sunburn after 30 to 40 minutes. Given that the average reported sun exposure of the participants is four hours, those with skin types I-IV are above the upper limit of exposure during the summer months [21].

Utilizing sunscreen and protective clothing can mitigate the damage associated with UV exposure and should be explored as a modifiable behavior to decrease skin cancer in this population. While sunscreen use is the most common protective measure employed by Americans, using sunscreen ranked least common among the protective practices utilized by the survey participants. This echoes the findings from a 2015 survey of uninsured primary care patients at a free clinic [14,22]. Instead, protective clothing, including hats and short/long-sleeved shirts proved to be most common, with an average response about their usage ranging from “sometimes” to “often.” These results demonstrate that individuals from low-income groups tend to use different protective measures than the general public, possibly due to the limited availability of sunscreen. Distributing sunscreen at local clinics and shelters to address this gap could be an effective intervention if awareness campaigns are run to encourage the utilization of these resources. Free clinics may also opt to supply interventions that may be more likely to be used in this patient population, such as UV protective clothing, hats, and sunglasses. 

Our study highlighted a concerning trend regarding the awareness and knowledge surrounding preventive measures for skin cancer and the identification of its indicators. Participants underwent assessment through six targeted inquiries focusing on optimal sunscreen application, recognition of skin cancer warning signs, and comprehension of the ramifications of tanning. On average, their accuracy in responding to these questions stood at three out of five questions (60%), signaling a prevalent deficiency in the knowledge about skin cancer prevention among the surveyed population. This gap appears rooted in inadequate community education regarding the risks associated with skin cancer, compounded by limited access to dermatologic care. Merely 10 participants (20%) reported receiving dermatologist care. Dermatologist visits are an important opportunity for crucial patient education on skin cancer; our surveyed population may be experiencing access barriers in obtaining these services. This lack of knowledge may lead to underestimated self-perception, particularly noteworthy among the Black population, where there exists a lower awareness of the potential for individuals with darker skin tones to develop skin cancer compared to the perception among White individuals regarding their risk [15].

The lack of awareness regarding the perceived risk might significantly diminish the frequency of individuals seeking dermatological care, thus reducing the visits to dermatologists. While common barriers to seeking dermatological care like transportation limitations, insurance issues, and long wait times have been extensively discussed in literature, our study's participants did not identify these factors as primary barriers. Rather, the most prevalent response was "none of the above," implying either an absence of perceived barriers or the presence of unmentioned obstacles.

In our study, only seven participants (14%) reported undergoing yearly skin checks, aligning closely with national statistics - merely 16% in men and 13% in women in the last year [23]. One study reported a similar frequency (13%) of skin checks amongst men residing at a homeless shelter in Texas [15]. Additionally, considering the average participant age in our study was 45.6 years and the typical onset age of skin cancer is 77 years, this demographic might not be at an age where access to dermatological care becomes a definitive concern [24].

The limitations of this study include the relatively small sample size, as well as factors common to research based on surveys, including response, non-response, and convenience sampling biases. The strengths of this study include the integration of a previously validated survey in a population experiencing homelessness as well as contribution to the literature on a traditionally underrepresented patient population. Moreover, sun safety practices among this predominantly homeless and underinsured population in the Midwest has not been studied before. While these results cannot be generalized, our findings may be beneficial to other free clinics and help guide services and modules for increasing sun safety activities in a way that is accessible and useful for this population. 

## Conclusions

Our study suggests a potential disconnect between the perceived risk, knowledge of skin cancer, and regular dermatological visits among the studied population. While traditional barriers like transportation and insurance were not highlighted, the prevalence of infrequent skin checks indicates a broader issue in recognizing the importance of regular dermatological care for early detection and prevention of skin cancer. The knowledge gaps surrounding sun safety suggest a potential area for targeted education and outreach. Moreover, our population's preferred sun safety protection methods highlight ways free clinics can best support patients - by providing access to sun protection that is feasible and usable by this population. A multifactorial approach involving awareness, access, and screenings would be valuable at this site.

## Appendices

1. Do you feel that you sunburn easily? (demographic)

a. Yes

b. No

2. In the summer, on average, how many hours are you outside per day between 10am and 4pm? (behaviors)

a. 30 minutes or less

b. 31 minutes to 1 hour

c. 2 hours

d. 3 hours

e. 4 hours

f. 5 hours

g. 6+ hours

3. Have you ever seen a dermatologist before for any reason? (barriers)

a. Yes

b. No

4. Have you ever had your skin checked head to toe for skin cancer by a dermatologist or other health professional? (barriers)

a. Yes

i. If yes, what year did you have your last skin check? _______

b. No

5. What is the most significant barrier you have encountered in obtaining dermatologic care? (barriers)

a. Transportation limitations.

b. Insurance/cost concerns.

c. Long wait times for appointments.

d. None of the above.

6. When should sunscreen be applied for best protection? (knowledge)

a. Just before going in the sun.

b. 15-30 minutes before going in the sun.

c. Within 15-30 minutes after going in the sun.

7. How often should SPF 30 sunscreen be reapplied? (knowledge)

a. Every 30 minutes.

b. Every 2-3 hours and more often if sweating or swimming.

c. Neither A nor B.

8. Which of the following could be a sign of skin cancer? (knowledge)

a. A sudden or gradual change in a mole’s appearance.

b. A sore that does not heal.

c. Both A and B.

d. Neither A nor B.

9. Please rank how often you perform the following behaviors when you go outside in the summer between 10am to 4pm. (behaviors)

**Table 4:** Likert scale behaviors for question nine in the survey.

Behaviors	Never (0% of the time)	Rarely (25% of the time or less)	Sometimes (about 50% of the time)	Often (75% of the time or more)	Always (100% of the time)
How often do you wear sunscreen?					
How often do you wear a shirt with sleeves that cover your shoulders?					
How often do you wear a hat?					
How often do you stay in the shade or use an umbrella?					
How often do you wear sunglasses?					
How often do you wear clothing made with specific sun protective material (fabric with SPF capabilities)?					

10. Please indicate whether you believe the following statements are true or false. (knowledge)

**Table 5:** True/false statements for question 10 of the survey. Correct answers are indicated by X.

Statement	True	False
Having a tan protects my skin from the sun.		X
A tan is a sign that the skin is damaged.	X	
A sunscreen with a SPF of 30 means a person can stay in the sun 30 times longer without burning than they could if not wearing sunscreen.	X	

11. Please indicate your degree of agreement with the following statements. (attitudes)

**Table 6:** Likert-scale statements for question 11 in the survey

Statement	Strongly disagree	Somewhat disagree	Neither agree nor disagree	Somewhat agree	Strongly agree
I am at risk for skin cancer.					
Sun protection is important.					
I have adequate access to sun protective items.					
Skin cancer is dangerous.					

12. What is your age? ___________ (demographic)

13. What is your race? (demographic)

a. White

b. Black

c. Asian

d. Other

14. What is your ethnicity? (demographic)

a. Not Hispanic/Latino

b. Hispanic/Latino

15. What is your insurance status? (demographic)

a. Uninsured

b. Medicare

c. Medicaid

d. Private insurance/employer-paid

16. What is your housing status? (demographic)

a. Own/rent my home

b. Live with friends/family

c. Live at a shelter or temporary housing

d. Homeless or no fixed residence
